# Advancements in Biomedical and Bioengineering Technologies in Sports Monitoring and Healthcare

**DOI:** 10.3390/bioengineering11080816

**Published:** 2024-08-12

**Authors:** Yaodong Gu, Justin Fernandez

**Affiliations:** 1 Department of Radiology, Ningbo No. 2 Hospital, Ningbo 315211, China; 2 Faculty of Sports Science, Ningbo University, Ningbo 315211, China; 3 Auckland Bioengineering Institute, Bioengineering Institute, Auckland 1142, New Zealand; j.fernandez@auckland.ac.nz

The intersection of biomedical and bioengineering technologies with sports monitoring and healthcare has recently emerged as a key area of innovation and research. The integration of these technologies promises to address long-term challenges in the fields of sports monitoring and healthcare. In the past, traditional sports monitoring approaches have struggled to meet their individualized demand; generalized training plans may reduce training effectiveness and increased injury risk [1]. Similarly, the healthcare sector faces significant challenges, including managing chronic diseases, the need for continuous patient monitoring, and the limitations of conventional diagnostics [2].

In modern times, wearable devices equipped with sensors can track various physiological parameters, such as heart rate, oxygen levels, and muscle activity [3,4,5]. These data can be used to customize training programs and tailor them to an individual’s needs, optimizing performance and significantly reducing the risk of injury. In healthcare, these current technologies are leading to significant improvements in patient care. Particularly, telemedicine platforms break down geographical barriers, providing access to healthcare services for remote and underserved populations [6,7]. Additionally, smart prosthetics and advanced rehabilitation devices enhance the quality of life of patients with physical disabilities [8,9]. These applications highlight the immense potential of biomedical and bioengineering technologies to provide innovative solutions to challenges in the field of sports monitoring and healthcare. 

This Special Issue showcases the latest research and advances in the application of biomedical and bioengineering technologies in sports monitoring and healthcare. It seeks to explore how to use these innovative technologies to address challenges in sports monitoring and healthcare and create more personalized, efficient, and effective solutions. 

Data mining is a critical method in biomedical and bioengineering technology, allowing the extraction of meaningful patterns and trends from large datasets, enhancing the effectiveness of sports monitoring and healthcare [10,11]. In this Special Issue, Jiang et al. [12] explores the biomechanical differences between novice and experienced runners following a 5 km run. Their study used principal component analysis to highlight that novice runners show greater changes in joint angles, joint moments, and ground reaction forces, which could be linked to a higher risk of lower-limb injuries. These findings are crucial for distinguishing runner levels and monitoring performance and injury risk. Additionally, advanced imaging and motion analysis technologies in sports science can help to deepen our understanding of mechanisms affecting running performance and injury risk, thus improving performance and preventing injuries [13,14]. Li et al. [15] use an ultrasonography system, revealing that long-term running with a forefoot strike pattern significantly affects the muscle fascicle length and pennation angle of the medial gastrocnemius, indicating that this strike pattern could lead to a longer fascicle length and a smaller pennation angle, which may protect the medial gastrocnemius from strain under repetitive high loads. Liu et al. [16] examined how different running velocities and shoe stiffness levels affect joint kinematics and asymmetry, which is critical for understanding running biomechanics and informing the design of smarter sports equipment. Cen et al. [17] compared foot–ankle temporal kinematics during planned and unplanned gait termination in subjects with different arch stiffnesses using statistical nonparametric mapping, providing insights into the compensatory adjustments of lower-limb joints during gait termination.

Biomechanical modeling and simulation, including finite element analysis (FEA) and musculoskeletal modeling, offer essential insights into the mechanical behavior of biological tissues. These technologies aid in designing medical devices, prosthetics, and injury prevention strategies, enhancing human movement understanding and rehabilitation program effectiveness. In this Special Issue, Henriques et al. [18] presented the Efficient Neck Model—2D (ENM-2D), a computationally efficient model designed to simulate a whiplash injury mechanism. Wang et al. [19]. focused on a detailed three-dimensional (3D) finite element (FE) model of the head–neck complex (C0–C7) to analyze its kinematics under rear-end impact conditions. These two studies offer theoretical support and practical tools for predicting and preventing whiplash injuries, advancing vehicle safety technology. Pan et al. [20] used FEA to evaluate the biomechanical effects of three different pedicle screw fixation methods (bilateral, unilateral, and lateral) combined with oblique lumbar interbody fusion surgery. Their study reveals that the total deformation and endplate stress of a unilateral pedicle screw fixation are comparable to bilateral fixation, offering spine surgeons a viable alternative that may reduce surgery time. Musculoskeletal modeling integrates anatomical and physiological data to simulate human movement mechanics, facilitating the understanding of movement disorders and the development of rehabilitation protocols. Cassiolas et al. [21] created a multibody musculoskeletal model with a customizable frontal knee alignment capable of estimating tibiofemoral contact forces during the execution of highly dynamic movements. However, improving the accuracy of musculoskeletal modeling to bring the simulation increasingly closer to a real-life scenario is something that researchers have been pursuing recently. Nasseri et al. [22] investigated the consequences of limiting electromyography and ground reaction force (GRF) data on model-estimated ACL force. They found that simplifying muscle activation patterns and reducing 3D GRF to vertical GRF results in spurious model estimates, underscoring the need for comprehensive data assessing ACL loading during dynamic tasks.

Advanced materials and wearable devices also significantly contribute to this research field. Jayathilaka et al. [23] found that a flexible and wearable graphene heating device has the potential to accelerate fatigue recovery, this device positively impacts fatigue recovery and muscle function, suggesting its potential in sports and rehabilitation settings. The Intelligent Internet of Medical Things (IIoMT) offers significant advantages in tracking treatment outcomes by providing real-time, continuous monitoring of patient health data. In their study, Pal et al. [24] rely on cardiac signals, which follow an integrated detection–estimation–reduction framework for anxiety using the Intelligent Internet of Medical Things and Mano Shakti Yoga, the latter of which the study suggests as an evidence-based exercise modality. The advent of advanced wearable devices and sensor technologies is transforming sports monitoring by providing real-time, personalized feedback, optimizing training regimens, enhancing athletic performance, and minimizing injury risk. Biomedical engineering approaches are an important part of addressing healthcare challenges. Innovations such as wearable health monitors, telemedicine solutions, and smart prosthetics are revolutionizing patient care. These technologies enable continuous health monitoring, early disease detection, and timely intervention, ultimately improving patient outcomes and reducing healthcare costs.

This Special Issue highlights the potential of biomedical and bioengineering technologies in the fields of sports monitoring and healthcare. As these fields continue to advance, we can look forward to achieving more sophisticated and integrated solutions that will further enhance personalized care, improve sports performance, and reduce the risk of injury. Future developments may include more advanced wearable technologies, smarter prosthetics, and innovative rehabilitation programs, all driven by data and tailored to individual needs. By embracing these innovations, we will move towards a future where healthcare and sports performance are not only optimized, but also more accessible and effective for everyone.
